# Advances in the microbiological diagnosis of herpetic retinitis

**DOI:** 10.3389/fopht.2022.990240

**Published:** 2022-09-13

**Authors:** Julie Gueudry, Bahram Bodaghi

**Affiliations:** ^1^ Department of Ophthalmology - Charles Nicolle University Hospital, CHU Charles Nicolle, Rouen, France; ^2^ Department of Ophthalmology, DHU ViewRestore, Sorbonne Université, Pitié Salpêtrière Hospital, Paris, France

**Keywords:** uveitis, retinitis, herpesvirus, PCR, ocular sample

## Abstract

Viral retinitis associated with herpesvirus is one of the most severe forms of uveitis and is a potentially sight-threatening ophthalmologic disease. The prognosis is poor and a rapid and aggressive management is necessary to improve the visual and sometimes vital prognosis of these patients. The treatments used are not without side effects, while many differential diagnoses exist, such as toxoplasmic retinochoroiditis, syphilitic retinitis, endogenous endophthalmitis and intraocular lymphoma. Causatives viruses are herpes simplex virus, varicella-zoster virus, and cytomegalovirus, which require rapid detection in ocular fluid, mainly aqueous humor. However, only a small amount of intraocular fluid is available for analysis. Advances in microbiological diagnostic techniques therefore were key factors in improving the management of these diseases. Historically, the diagnosis was based on immunological tests but more recently advances in molecular biology, in particular polymerase chain reaction, have played a crucial role to obtain a reliable and rapid diagnosis of viral retinitis associated with herpesvirus, as discussed in this review.

## 1. Introduction

Viral retinitis includes many clinical presentations, varying according to the immune status of the host. Human herpesviruses are the most frequent causes of viral posterior uveitis. The viruses involved are herpes simplex virus type 1 et 2 (HSV1, HSV2), varicella-zoster virus (VZV), cytomegalovirus (CMV) and for some authors Epstein–Barr virus (EBV), which are double-stranded DNA viruses, able to establish latency after primary infection (1). Herpetic retinitis includes acute retinal necrosis (ARN), progressive outer retinal necrosis (PORN), CMV retinitis, and non-necrotizing retinopathy. Herpetic retinitis is rare but sight threatening. Prognosis depends mainly on rapid diagnosis and prompt initiation of appropriate treatment, and nothing should delay the initiation of antiviral therapy in case of clinical suspicion. Nevertheless, required treatments are long and could have side effects, in particular nephrotoxic or hematotoxic effects; furthermore, differential diagnoses are multiple and may sometimes lead to difficulties in clinical diagnosis. Therefore, a rapid and reliable diagnosis is necessary, with the limitation of small sample volumes and the need to analyze several viruses or microorganisms that may be involved. Over the past 20 years, the application of molecular biology to intraocular samples has revolutionized the management of these patients. These biologic data have been progressively included in revisited diagnostic criteria. This review aims to describe recent advances in the microbiologic diagnosis in different herpetic retinitis. We conducted a narrative review by selecting articles written in English and French from PubMed/MEDLINE database published until June 2022. The keywords used to screen the database were searched in MeSH (Medical Subject Headings) and were: (acute retinal necrosis) AND (diagnosis) and (viral) OR (HSV) OR (VZV) OR (CMV) OR (herpetic) AND (retinitis) AND (diagnosis).

## 2. Herpetic retinitis

### 2.1 Acute retinal necrosis syndrome

**Figure 1:**
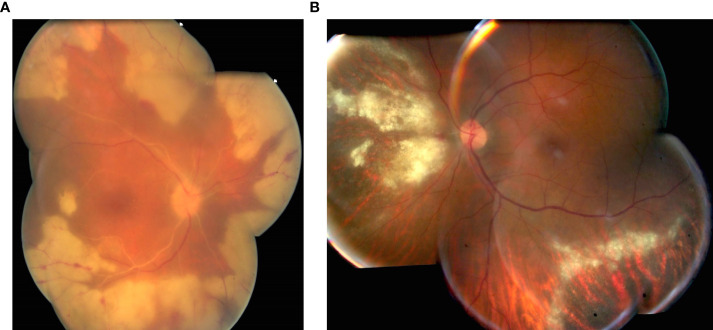
**(A)** Severe acute retinal necrosis syndrome associated with mild vitritis. PCR analysis of aqueous humor was positive for VZV; **(B)** Granular form of CMV retinitis in a severe immunosuppressed patient diagnosed by PCR analysis in aqueous humor.

Acute retinal necrosis was first described in 1971 by Urayama *et al.*, as unilateral panuveitis with retinal vasculitis leading to diffuse retinal necrosis and retinal detachment (**Figure 1A**) (2). It is a rare condition. Two studies in the UK estimated it to be one case per 1.6 to 2.0 million inhabitants per year (3, 4). ARN is typically found in middle-aged patients (5). HSV-2 retinitis occurs in younger patients while HSV-1 or VZV retinitis occurs later (6). Even though EBV has been suggested to induce clinical features of ARN, these data are still uncertain (7). Although, the majority of ARN cases were predominantly unilateral at presentation, diseases could become bilateral in the absence of appropriate early treatment (8).

Holland et al. defined diagnostic criteria in 1994 for the American uveitis society associating well-demarcated areas of retinal necrosis in the peripheral retina, rapid circumferential progression of retinal necrosis, occlusive vasculopathy, and a prominent intraocular inflammatory reaction (9). In 2015, Takase *et al.* attempted to establish new diagnostic criteria, integrating the results of intraocular sample analysis (10). In 2021, diagnostic criteria were published by the standardization of uveitis nomenclature (SUN), emphasizing the need to isolate the causative agent, without making it mandatory, to diagnose ARN at the first observation by removing the criterion of progression, and modulating the clinical presentation according to the immune status. Recent SUN criteria are the presence of necrotizing retinitis involving the peripheral retina AND evidence of infection with either HSV or VZV i.e. positive PCR from either an aqueous or vitreous specimen OR presence of characteristic clinical picture such as circumferential or confluent retinitis and retinal vascular sheathing and/or occlusion and more than minimal vitritis in immunocompetent patients. Exclusion criteria are positive serology for syphilis and intraocular specimen PCR-positive for CMV or *Toxoplasma gondii* (7).

### 2.2 Progressive outer retinal necrosis syndrome

Progressive outer retinal necrosis syndrome was first described in 1990 (11). The evolution of PORN is fulminant with poor prognosis, occurring in severely immunosuppressed patients. PORN syndrome is most often caused by VZV and is bilateral in onset. Painless, minimal intraocular inflammation is associated with deep multifocal retinal necrosis, early posterior pole involvement, without retinal vasculitis, which progress rapidly (12). In the era of modern antiretroviral therapy, the current incidence rate of PORN is very low, difficult to estimate (13).

### 2.3 Cytomegalovirus retinitis

The development of highly active antiretroviral therapy (HAART) in the mid 1990’s profoundly changed the natural history of CMV retinitis in HIV-positive patients (14). The remaining risk factor for CMV retinitis is iatrogenic immune compromise particularly in case of organ or bone marrow transplant (15). The pathogenesis of CMV retinitis among immunosuppressed patients is suspected to be reactivation of a latent infection followed by hematological dissemination (16). In immunocompetent patients, CMV retinitis was described following intraocular corticosteroid injections (17). The fulminant form is associated with extensive areas of necrotizing retinitis and hemorrhage. The granular form is more indolent associated with granular and grayish retinal edema (**Figure 1B**) (15). Frosted branch angiitis was associated with CMV retinitis (18). Most often, there is no or mild vitritis indicating immunosuppression. SUN criteria are necrotizing retinitis with indistinct borders due to numerous small satellites AND systemic or ocular Immune compromise e.g. AIDS, chemotherapy, organ transplant or intraocular chemotherapy or corticosteroids respectively AND presence of characteristic clinical picture as wedge-shaped area of retinitis or hemorrhagic appearance of the retinitis or granular appearance of the retinitis without or with mild vitritis OR positive PCR for cytomegalovirus from either the aqueous or vitreous specimen (16).

### 2.4 Non-necrotizing herpetic retinopathies

This nosological entity was described through advances in virological diagnostic techniques. Bodaghi et al. showed that amplification of DNA from aqueous humor was sensitive enough to detect causative agents of non-necrotizing herpetic retinopathies in case of atypical forms of uni- or bi-lateral corticosteroid-resistant posterior uveitis. HSV-1 and VZV were identified. Patients presented chronic and longstanding inflammation including vitritis, vascular leakage visible on fluorescein angiography, and retinal edema, without retinal necrosis (19). Since then, several cases have been reported, mainly retinal vasculitis (20–25).

## 3. Virological diagnosis

### 3.1 Background

Historically, the diagnosis of herpetic retinitis was based on clinical examination and was confirmed with a favorable response to antiviral treatment. In 1982, Culberston et al. suggested for the first time a viral cause to ARN after histopathologic and electron microscopy analysis of an enucleated eye (26). Since this first report, the viral cause has been confirmed by direct and indirect methods. Among direct methods used, There were analysis of an endo-retinal biopsy with electron microscopy, without however being able to distinguish which herpesvirus was involved (27), VZV and HSV viral cultures from vitreous specimens (28–30), or immunocytochemical methods (31). Indirect methods were used such as detection of specific intraocular anti-antibody production (32–37). A concomitant occurrence between acute retinal necrosis and herpetic dermatitis, especially in case of varicella zoster dermatitis was also used (38, 39). In 1991, *Fox et al.* (40) detected CMV genome by PCR in intraocular fluids, i.e., aqueous humor (AH), vitreous, and subretinal fluid of patients with clinical features of CMV retinitis. This diagnostic method has changed the practical approach of intraocular infectious diseases (36, 37, 41–45).

### 3.2 Local antibody production analysis

The value of serum antibody testing is limited in the context of herpesvirus infections: nearly half of adults in France have serum antibodies to CMV (46), two-thirds to HSV-1 (47), and more than 90% to VZV (48, 49). Ocular complications often occur at a distance from the primary infection. Even though a negative serological test result could rule out a viral etiology of uveitis, false negatives are reported (50).

Ocular antibody production is often evaluated by comparing levels of specific and total IgG in AH and in serum. This obtained quotient of local antibody production is named Goldmann-Witmer coefficient (GWC). Specifically, local antibody production of anti-herpes virus antibodies is measured by the quotient: (titer against herpes antigens in the vitreous or AH/IgG amount in the vitreous humor or AH)/(antibody titer against herpes antigens in the serum/IgG amount in the serum) (33, 51). Values above 3 are considered as positive (52). It should be performed between 2 weeks and 3 months of evolution to improve diagnostic performance. However, the rate of false negatives in case of immunosuppression may be due to a low production of antibodies in the AH (53). False negativity can also be linked to intraocular sample collected too early, i.e., when local production is not yet detectable, or to a rupture of the blood-retinal and aqueous barriers leading to a ratio close to 1. In this last situation, Quentin et al. proposed a correction factor to improve reliability (54). The other disadvantage lies in the need for a large amount of AH. Intraocular specific antibodies production can be analysed from 40 to 100 μl of ocular fluid (55, 56).

Immunoblotting can help to recognize intraocular specific antibodies from smaller specimen i.e. 10 µl. This procedure has been developed in ophthalmology diagnosis for ocular toxoplasmosis (57–59), and more recently to diagnose ocular syphilis (60).

### 3.3 Polymerase chain reaction

#### 3.3.1 Efficacy

Polymerase chain reaction (PCR) is a direct method to detect viral DNA based on enzymatic amplification of nucleic acids with specific primers and a thermoresistant DNA polymerase. It is a highly sensitive method for the detection of the viral genome. In the context of suspected ARN, PCR analyses were positive for VZV or HSV in 79 to 100% of cases (61). Furthermore, PCR requires only a small amount of intraocular fluid (6) and is a rapid diagnostic technique. In the absence of clinical signs in favor of viral retinitis, the genome of herpes group viruses was undetectable by PCR despite previous immunization (62). An initial negative result and a strong clinical suspicion should lead to repeated anterior paracentesis and/or to consider another diagnosis.

#### 3.3.2 Different PCR techniques

**Figure 2:**
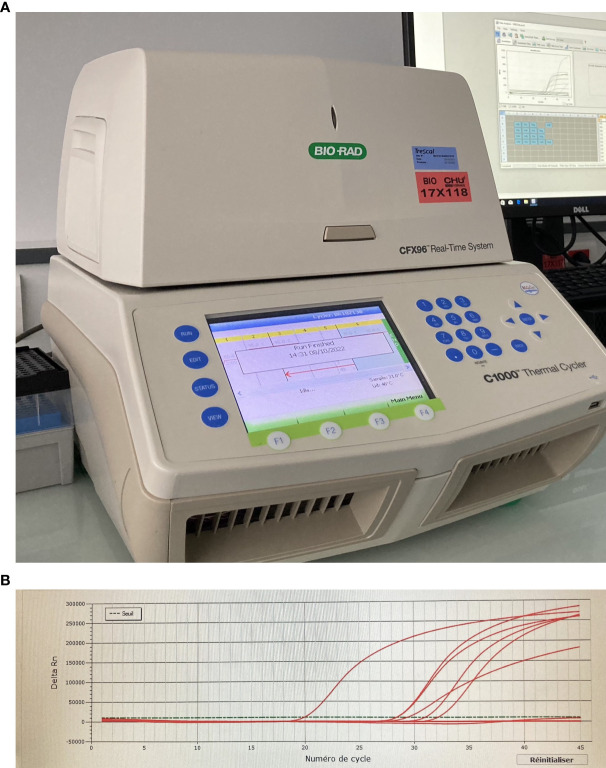
**(A)** Thermal cycler or PCR machine; **(B)** Representative graph of multiplex real-time PCR results for HSV1. The fluorescence intensity at each cycle is proportional to the concentration of amplicons, statistically different from the baseline when higher than the threshold. (seuil: threshold; numéro de cycle: cycle number).

PCR has been used in ophthalmology diagnosis for 20 years (63). At the beginning, the detection and analysis of amplified DNA products were realized by agarose gel electrophoresis. The entire process lasted 4-5 hours to complete and only a limited number of viruses could be tested. More recently, multiplex qualitative PCR combines several different primer pairs in the same reaction and can test multiple viruses simultaneously. A qualitative multiplex PCR is a useful tool to detect and screen intraocular herpesvirus infections, even when only a small amount of intraocular fluid is available. Sugita et al. described a multiplex PCR designed to qualitatively analyze DNA of eight human herpesviruses. However, authors explained that quantitative real-time PCR was mandatory to confirm the clinical relevance of these multiple PCR results (64). Real-time PCR allows continuous monitoring of the PCR amplification process by detecting the fluorescence emitted by the newly formed PCR products (**Figure 2**) (65).

A recent multiplex real-time PCR prototype, named Strip PCR, was designed to detect 24 viral, bacteriological, fungal and parasitological pathogens (66). This Strip PCR included 12 precoated wells. The reagent was solidified to obtain a simpler and faster test. However, this prototype was limited by contaminations and was modified to detect a smaller number of 9 pathogens including 6 herpesviruses. This technique was evaluated in a multicenter study using Strip PCR and showed high sensitivity and specificity in 772 intraocular samples (67). The same team developed a simpler PCR without the DNA purification step (63).

For each PCR reaction, 10-20 µl of ocular fluid were used (68). Currently, multiplex PCR can test simultaneously multiple viruses from similar amount of ocular specimen from 25 to 50 µl (67, 69).

### 3.4 Next-generation sequencing technology

Next-generation sequencing (NGS) technology can provide a large quantity of DNA or RNA very rapidly from a small amount of sample. Its use in the clinic is constantly increasing despite the challenges of data interpretation. This technique allows, in virological diagnosis, to identify viruses that were not suspected in the initial diagnosis or even novel viral pathogens. It can also identify different populations of causative viruses that would not have been identified with the classical method of PCR (70). The use of NGS in ophthalmologic microbiological diagnosis is currently still limited (71). It has been used in the diagnosis of intraocular infection to identify a viral cause of severe refractory uveitis (72, 73). It has also been used in the diagnosis of lacrimal sac infections and microbial keratitis (74, 75). Its use in the diagnosis and management of viral retinitis has not yet been reported. Nevertheless, we can presume that this technology will be part of routine techniques in the near future, identifying infectious agents and their heterogeneity.

### 3.5 Local antibody production analysis and PCR: Complementary tests?

PCR techniques and herpesvirus-specific ocular antibody production analysis are techniques that appear complementary because some cases not diagnosed by PCR could be diagnosed with ocular antibody production analysis, particularly during the chronic phase of uveitis even in case of low anterior chamber inflammation. Bojanova et al. described retrospectively the ocular production of specific antiviral IgG in 32 out of 62 (51.6%) herpesvirus PCR-positive uveitis cases (51.6%), none in the 42 control patients, and in 21 out of 38 (55.2%) of PCR-negative cases highly suspected to harbor herpesvirus uveitis. In this study, anterior and posterior uveitis were studied, i.e., 55 cases of anterior uveitis with positive PCR and 7 cases of ARN with positive PCR (56). A different pattern for viral infection and ocular toxoplasmosis was described, with a preponderant role of molecular biology in herpesvirus ocular infections and conversely, a preponderant role of immunological diagnosis in the case of ocular toxoplasmosis. In viral infection, PCR was positive early in the disease, whereas PCR was negative at later stages and GWC was positive (69).

A recent review compared sensitivity of PCR and GWC for the diagnosis of viral uveitis. Among published data, sensitivity was estimated from 79% to 100% for PCR and 57% for GWC in ARN; up to 100% for PCR and limited utility due to occurrence in severe immunosuppressed for GWC in PORN; and from 93% to 95% with a rapid decrease after initiation of treatment for PCR and limited utility due to occurrence in severe immunosuppressed for GWC (21%) in CMV retinitis (76). Based on the immune status of the host, clinical setting, delay between sampling and onset of disease, and the small amount of intraocular fluid available, it is most often necessary to choose between PCR or GWC for the first analysis, even though it is better to perform both. In the context of viral retinitis, PCR is most often performed first line.

### 3.6 Where to sample?

To obtain ocular fluids, anterior chamber paracentesis (ACP) is a safe procedure and up to 0.2 mL can be obtained. In a retrospective study including 361 patients, hyphema was reported in seven cases, but no serious complications such as cataract, keratitis, or endophthalmitis were reported (77). Nonetheless, it should be considered as a surgical procedure. Diagnostic vitrectomy for vitreous fluid is more complex than ACP but provides a larger amount of fluid. Retinal detachment remains a complication of vitreous biopsy or vitrectomy but its incidence remains low (41); one out of 44 patients in a recent case series (78). Nonetheless, cataract development must be considered as a frequent complication as reported in a half of 65 patients by Oahalou et al. (79). If intraocular sampling is problematic, isolation of CMV from blood or urine can be considered, as previously reported (80, 81).

There is no clear difference between the detection rates of PCR performed from aqueous or vitreous samples (42). Only one study reported in ARN diagnosis a difference in positive diagnosis rate, i.e., 93% positive for vitreous vs 46% positive for aqueous specimens; however, it was not a simultaneous comparative study (4). ACP may be safer, less invasive, and easier than vitreous biopsy.

## 4. Monitoring and resistance

### 4.1 Prognosis according to herpesvirus type

VZV-related ARN syndrome appears to be more severe, with sight-threatening outcomes. Wong et al. reported that patients with VZV ARN presented with poor final visual acuity (VA), i.e., ≤ 20/200 after a 12-month follow-up and 2.5-fold greater risk of retinal detachment compared with patients with HSV ARN, despite similar initial VA on presentation and no difference in the initial area of retinitis (82). In 2000, using PCR of the variable region R1, Abe et al. showed that different VZV strains were associated with ARN (83). Series reporting HSV2 ARN are rare. The prognostic difference between HSV-1 and HSV-2 ARN was not investigated directly in literature. HSV-2 ARN occurs predominantly in young or pediatric populations, which could delay diagnosis and thus change outcome (84).

A prompt start of systemic therapy could prevent involvement of the unaffected eye. Palay et al. showed, in a retrospectively study, that systemic acyclovir significantly decreased second eye involvement, 12.9% vs 69.9% between patients treated with acyclovir and not treated with acyclovir, respectively (85).

### 4.2 Herpesvirus resistance

Phenotypic resistance is determined by the ability of herpesvirus to grow in the presence of an anti-viral drug, usually expressed as half maximal inhibitory concentration (IC_50_). Genotypic resistance is determined by the detection of a mutation known to provide a resistant phenotype (86). The advantage of PCR is the rapidity of analysis compared with virological culture, i.e., less than 48 h vs. more than 4 weeks  (87). NGS technology is able to identify heterogeneous resistant viral populations and their adaptation to antiviral treatment (88).

Antiviral systemic treatment i.e. acyclovir is the first line of treatment for HSV- or VZV- retinitis, most of the time combined with intravitreal antiviral therapy i.e. foscarnet or ganciclovir. Clinical resistance of the herpesvirus to acyclovir may be suspected if there is a worsening of the disease during the first few days despite adequate treatment. In case of refractory disease or ocular resistance to acyclovir, intravenous foscarnet should be considered (89). Molecules with different mechanisms of action, not targeting DNA polymerase, are currently under development such as helicase-primase inhibitors for acyclovir-resistant HSV and VZV management (84), and HSV glycoprotein-specific antibody (90). Several molecules are approved antiviral drugs for CMV infection. Ganciclovir and valganciclovir are first line therapy. In case of resistance, foscarnet is the second therapy. Cidofovir use is limited due to its toxicity. In case of side effects or resistance, recent molecules can be used such as letermovir which is a CMV-terminase inhibitor (85–87).

It therefore seems useful to provide definitive virological confirmation of resistance to acyclovir. Tran et al., in 2005 described a presumed acyclovir- resistant HSV-2 in severe ARN in an immunocompetent 11-year-old boy. Firstly, HSV2 was isolated by PCR in the AH. Then, acyclovir-resistant HSV-2 was isolated by culture from forearm vesicules, which appeared during ocular disease treatment, while corticosteroids had just been introduced. Acyclovir resistance determination was based on the calculation of inhibitory concentrations. Furthermore, thymidine kinase gene was amplified by PCR and compared to the reference strain. A missense mutation was detected conferring acyclovir resistance. The therapeutic adaptation permitted the maintenance of functional visual acuity (91). Acyclovir resistance due to mutations in thymidine kinase gene in HSV-1 ARN and HSV-2 ARN were further reported based on PCR analysis (92, 93).

HSV and VZV genotypic resistance analysis is based on amplification and sequencing thymidine kinase and DNA polymerase genes to detect resistance-related mutations. Thymidine kinase gene (UL23) and DNA polymerase (UL30) genes can be amplified and then sequenced, as well as TK gene (ORF36) and DNA polymerase gene in VZV. Genotypic methods are faster than phenotypic ones, but are unable to analyze with precision the clinical consequences of all the mutations, in particular those not yet described and those not resulting in a stop-codon or a frameshift mutation (86). Moreover, the confirmation of herpesvirus resistance contributes also to prevent diagnostic errors as wrongly excluding the diagnosis of viral retinitis in the case of a poor therapeutic response.

The clinical impact of CMV-resistance to ganciclovir is well documented (94). Ganciclovir resistance mutations were identified in both UL97 gene coding for UL 97 phosphotransferase and UL54 gene coding for viral DNA polymerase. Cidofovir and foscarnet were only associated with UL54 mutations (95). Some studies highlighted the possible presence of different CMV genotypes in blood and AH in the same patient. These differences prompt the determination of a possible CMV drug-resistance from ocular samples instead of CMV isolated in blood, especially during antiviral treatment and follow up, even though the blood sequencing of CMV DNA remains clinically useful (96, 97).

### 4.3 Virological monitoring

More recently, quantitative real-time PCR has shown that it is also able to quantify the viral load, in order to establish prognostic factors and to guide treatment in patients with ARN. Abe et al. showed that a higher VZV DNA copy number in ocular fluids, estimated with PCR and semi nested PCR, was associated with worse visual acuity in 12 eyes out of 11 patients. Moreover, VZV DNA copy number tended to be higher in elderly and immunosuppressed patients associated with less efficacy of treatment (98). A quantitative DNA copy number of ≥5.0 × 106/mL in anterior chamber fluid was associated with more extensive retinitis, development of retinal detachment in patients with ARN syndrome and worse baseline and final VA in 14 eyes of 13 patients (99). Furthermore, case series analyzed the kinetics of viral DNA during retinitis treatment and suggested that quantitative PCR could be useful to monitor the response to antiviral drugs (100–102). Recently, in 17 patients a change in viral load during treatment was evaluated as 3 phases: an inconstant first plateau period, then a logarithmic decrease phase, and finally a negativation phase. Viral load monitoring could provide clues to antiviral resistance in case of prolonged initial plateau detection despite antiviral treatment (11.6 days vs 28 days) (92). Moreover, the level of CMV DNA in AH and vitreous was described to differentiate active and inactive CMV retinitis and to correlate with the surface area of active CMV retinitis (103).

## 5. Conclusion

In conclusion, advances in microbiological diagnostics have revolutionized the management of patients with retinitis associated with herpesviruses over the last few decades. Advances in techniques, particularly in molecular biology, have made it possible to improve the precision of rapid virological diagnosis, in a particular ophthalmological setting of very small sample volumes. The detection of viral resistance is now more easily achieved and has improved the ophthalmological management. The future will allow the development of even simpler and faster diagnostic methods for the reliable testing of all herpesviruses responsible for posterior uveitis, as well as the microbiological elements responsible for similar clinical feature in a single sample. The aim is to accelerate the initiation of appropriate treatment while limiting the side effects of other molecules started while waiting to exclude differential diagnoses.

## Author contributions

JG and BB prepared and revised the manuscript. The final manuscript version was approved by both authors.

## Acknowledgments

The authors are grateful to Nikki Sabourin-Gibbs (Rouen University Hospital) for her help in editing the manuscript and to virology department (Rouen University Hospital) for providing laboratory illustrations.

## Conflict of interest

The authors declare that the research was conducted in the absence of any commercial or financial relationships that could be construed as a potential conflict of interest.

## Publisher’s note

All claims expressed in this article are solely those of the authors and do not necessarily represent those of their affiliated organizations, or those of the publisher, the editors and the reviewers. Any product that may be evaluated in this article, or claim that may be made by its manufacturer, is not guaranteed or endorsed by the publisher.
